# Acute motor-sensory axonal polyneuropathy variant of Guillain–Barre syndrome complicating the recovery phase of coronavirus disease 2019 infection: a case report

**DOI:** 10.1186/s13256-021-02988-y

**Published:** 2021-07-16

**Authors:** Ahmed Maseh Haidary, Sarah Noor, Esmatullah Hamed, Tawab Baryali, Soma Rahmani, Maryam Ahmad, Farahnaz Erfani, Hashmatullah Azimi, Habib Ul Rahman Habib, Gul Ahmad Tahiri, Ramin Saadaat, Abdul Sami Ibrahimkhil, Esmatullah Esmat, Haider Ali Malakzai

**Affiliations:** 1 Department of Pathology and Laboratory Medicine, French medical Institute for Mothers and Children, Kabul, Afghanistan; 2 Department of Medicine, Jumhoriat Hospital, Afghan Ministry of Health, Kabul, Afghanistan; 3 Neurology Unit, Department of Medicine, French Medical Institute for Mothers and Children, Kabul, Afghanistan; 4 Department of Quality, French Medical Institute for Mothers and Children, Kabul, Afghanistan

**Keywords:** AMSAN, GBS, Recovery phase, COVID-19

## Abstract

**Introduction:**

The novel coronavirus, since its first identification in China, in December 2019, has shown remarkable heterogeneity in its clinical behavior. It has affected humans on every continent. Clinically, it has affected every organ system. The outcome has also been variable, with most of the older patients showing grave outcomes as compared with the younger individuals. Here we present a rare and severe variant of Guillain–Barre syndrome that complicated the disease in recovery phase.

**Case presentation:**

A 60-year-old Afghan man, who had been recovering from symptoms related to novel coronavirus associated disease, presented with sudden onset of progressive muscle weakness and oxygen desaturation. Electrophysiological workup confirmed the diagnosis of Guillain–Barre syndrome, and early institution of intravenous immunoglobulin resulted in complete resolution.

**Conclusion:**

Guillain–Barre syndrome has recently been reported in many patients diagnosed with novel coronavirus associated disease. While clinical suspicion is mandatory to guide towards an effective diagnostic workup, early diagnosis of this complication and timely institution of therapeutic interventions are indispensable and lifesaving.

## Introduction

Severe acute respiratory syndrome coronavirus 2 (SARS-CoV-2), which originated in Wuhan, Hubei Province, China in December 2019, has now become a global pandemic, devastating every corner of the world [1]. Almost every country in the world has been affected by the socioeconomic impact of the disease [2]. Many countries have imposed nationwide lockdown to tackle the disease spread that resulted in unconditional detrimental effect on international economy [3, 4].

Considering the clinical presentations, SARS-CoV-2-associated disease has shown remarkable heterogeneity in its clinical presentation [5, 6]. The wide spectrum of clinical features can be attributed to the fact that SARS-CoV-2 uses angiotensin II, the body’s most abundant receptor protein, as a portal to enter the cell [7]. The symptoms range from mere loss of taste and/or smell sensation with mild cough, usually in young adults, to severe and frequently life-threatening acute respiratory distress syndrome, mostly in older individuals [8]. In the context of classic clinical picture, devastating cardiovascular events have claimed many precious lives with formation of intracardiac thrombus complicating to embolic events [9]. Similarly, myocardial dysfunction and heart failure have been reported in many confirmed cases of coronavirus disease 2019 (COVID-19), in patients with previously normal hemodynamic status [10, 11].

Patients who presented with gastrointestinal symptoms had variable symptoms; whereas some patients developed acute gastroenteritis, others developed fulminant liver dysfunction [12]. Transient elevation in transaminase enzymes was a frequent sign of the disease [12].

Neurological abnormalities have been quite frequent in patients affected by COVID-19, ranging from peripheral nerve dysfunction to life-threatening encephalitis [13]. Many institutions have reported cases of Guillain–Barre Syndrome (GBS) in association with COVID-19. We present a case of AMSAN, a rare and severe variant of GBS, that affected a patient during recovery from COVID-19.

## Case presentation

A 61-year-old Afghan gentleman, businessman by profession, presented to a general practitioner with febrile illness associated with generalized body aches and pains, in early May 2020. He was a nonalcoholic and nonsmoker and had no significant past medical or surgical history. On examination, the patient was found to have normal oxygen saturation with normal physical examination findings. Thus, the patient was discharged home with paracetamol and was advised to maintain adequate hydration. During the first week of illness, the clinical progression was fairly controlled, but through the second and third weeks of illness, the patient developed malaise, loss of appetite, distaste, and loss of smell sensation. At that time, the patient’s oxygen saturation was still within normal range with normal hydration status, and the patient was thus continued with the original management plan and was asked to visit hospital if there was any aggravation of symptoms. Through the fourth week of illness, there was aggravation of clinical features with frequent drops in oxygen saturation noted overnight and generalized weakness, and cramps especially in the lower limbs. By the end of the first month of illness, the patient was afebrile with resolution of most of the symptoms including improvement of appetite and return of sense of smell and taste, but the body aches and muscle weakness further aggravated. This was when serological tests were performed for SARS-CoV-2 that showed strong IgG positivity with negative reaction for IgM, presented in Table 2. The patient had normal d-dimer, elevated C-reactive protein (23 mg/L), normal liver enzymes, and normal renal profile.

As presented in Table 1, in a span of 5 days, the patient’s condition deteriorated with development of flaccidity in all of the upper and lower limb muscles, demonstrating 1/5 power at shoulders and wrist joints on both sides and 2/5 power at hip and knee joints on both sides. Oxygen saturation also deteriorated with the lowest recorded value of 76% saturation. The laboratory workup revealed normal liver and renal profile with normal fasting blood glucose and blood counts, while arterial blood gas analysis revealed type II respiratory failure, as presented in Table 2. Thus, the impression given by consultant neurologist was Guillain–Barre syndrome, and immediate nerve conduction studies were performed. The nerve conduction studies, as presented in Tables 3 and 4, revealed diffuse axonal sensory-motor polyneuropathy. Considering the clinical context, the impression was acute sensory-motor axonal neuropathy (AMSAN), a variant of Guillain–Barre Syndrome (GBS). Accordingly, the patient was started on intravenous immunoglobulin (IVIG) with a dose of 400 mg per kilogram body weight daily, for 5 days. Following the commencement of IVIG, there was significant clinical alleviation of symptoms with improvement in respiratory functions, oxygen saturation, and return of muscle power, recovering to 4/5 power in the upper and lower limbs on both sides. Two weeks post IVIG therapy, with concomitant daily physiotherapy, the patient was able to be independent in active daily life, had muscle power of 5/5 at all the joints, and continued to maintain good health at the second month and then 6 months after initial diagnosis of GBS. At the moment, 9 months after the initial presentation, the patient is absolutely normal.

**Table 1:** Muscle power scale before and after IVIG therapy

Muscle	Power before IVIG therapy	Power 48 hours after IVIG therapy
Shoulders	1/5	4/5
Wrists	1/5	4/5
Hips	2/5	4/5
Both knees	2/5	4/5

**Table 2:** Laboratory investigations performed at the time of hospital admission

Laboratory investigations			
Parameters	Values	Parameters	Values
Complete blood count**		Biochemistry**	
Hb	14.8 g/dL	Liver function test**	
HCT	47.38%		
WBC	16,900/μL	AST	21 IU/L
Neutrophil	82%		
Lymphocyte	11%	ALT	11 IU/L
Eosinophil	0.5%		
Monocyte	6%	Total bilirubin	8 μmol/L
Basophil	0.5%		
ABG		Direct bilirubin	6.9 μmol/L
pH	7.21		
PCO_2_	73 mmHg	Indirect bilirubin	2.1 μmol/L
PO_2_	54 mmHg		
HCO_3_	31 mmHg	Renal function test**	
SPO_2_	84%	BUN	5 μmol/L
COVID-19 serologic test*		Creatinine	72 μmol/L
IgM	Negative	HbA_1C_**	5.1%
IgG	Positive	FBS**	4.1 mmol/L

*FBG* fasting blood glucose, *HBA1c* hemoglobin A1c, *CREAT* serum creatinine, *BUN* blood urea nitrogen, *ALT* serum alanine aminotransferase, *AST* serum aspartate aminotransferase, *TBILL* total bilirubin, *CBC* complete blood count, *Hb* hemoglobin, *WBC* total white blood cell count, *PLT* platelet count, *N* percentage of neutrophils, *L* percentage of lymphocytes, *E* percentage of eosinophils, *M* percentage of monocytes, *PH* power of hydrogen ion concentration, *PCO*_*2*_ partial pressure of carbon dioxide in blood, *PO*_*2*_ partial pressure of oxygen in blood, *HCO*_*3*_^*-*^ bicarbonate ion concentration, *SPO*^*2*^ oxygen saturation

*The test was done at the end of the fourth week of disease.

**The test was done during the first week of the second month of disease, during the hospital admission

**Table 3:** Motor nerve conduction studies

Nerve–muscle stimulus site	Stimulus site	Latency (milliseconds)		Distance (cm)		Amplitude (mV)		NCV (m/second)		F-LAT (milliseconds)		Duration (milliseconds)	
		Right	Left	Right	Left	Right	Left	Right	Left	Right	Left	Right	Left
Median—APB	Wrist		5.7		7.0		6.2						
Median—APB	Elbow		9.8		22.0		5.6		54.0				
Ulnar—ADM	Wrist		3.3		7.0		6.2				34.3		
Ulnar—ADM	B.Elbow		7.5		21.0		5.5		50.0				
Ulnar—ADM	A.Elbow		9.3		10.0		4.9		56.0				
Tibial—AHL	Ankle		7.2		10.0		1.3						
Tibial—AHL	Popliteal fossa		19.6		40.0		0.9		32.0				
Peroneal—EDB	Ankle		6.2		7.0		0.7						
Peroneal—EDB	Fibula head		13.5		29.0		0.8		40.0				
Peroneal—EDB	Popliteal fossa		15.6		8.0		0.7		38.0				

*APB* abductor pollicis brevis, *ADM* abductor digiti minimi, *B.Elbow* below elbow, *A.Elbow* above elbow, *AHL* abductor hallucis longus, *EDB* extensor digitorum brevis, *F2* finger 2, *F5* finger 5, *NR* not recorded, *NCV* nerve conduction velocity

**Table 4:** Sensory nerve conduction studies

Nerve	Recording site	Stimulating site	Latency (seconds)		Distance (cm)		Amplitude (μV)		NCV (m/second)	
			Right	Left	Right	Left	Right	Left	Right	Left
Median	F2	Wrist		NR		13.0		NR		NR
Ulnar	F5	Wrist		NR		11.0		NR		NR
Sural	Ankle	Calf		NR		14.0		NR		NR

*NCV* nerve conduction velocity, *F2* finger 2, *F5* finger 5, *NR* not recorded

## Discussion

Guillain–Barre Syndrome (GBS) is an acute inflammatory polyradiculoneuropathy resulting in flaccid paralysis in affected muscles [14]. The signs and symptoms can range from proximal muscle weakness to life-threatening respiratory failure [15]. In our patient, COVID-19-related signs and symptoms evolved over a period of 4 weeks. Although by the end of the month there was resolution of all the symptoms, the patient rapidly progressed to develop GBS-related flaccid paralysis during the recovery phase of the disease with significant clinical improvement upon administration of IVIG. It is worth mentioning that our patient presented with AMSAN, the severe variant of GBS, complicating the recovery phase of COVID-19, which was so far not been reported in the literature.

According to an estimate, more than 100,000 people are affected by GBS every year, worldwide [14]. The pathophysiological mechanism involved in the development of GBS is complex, but complement-mediated injury to the myelin sheath of peripheral nerves was identified to be the most common mechanism [16]. Complement-fixing anti-glycolipid autoantibodies target the nodes of Ranvier, which is the principle target in GBS [16]. Although such autoantibodies are the most pathogenic factors in the development of GBS, one-third of patients with GBS demonstrate no evidence of antibody development [17, 18].

GBS is triggered by a prior infection that initiates the pathogenic cross-reactive immune response, mostly in the form of autoantibodies [16, 20]. Infection by *Campylobacter jejuni*, cytomegalovirus, Epstein–Barr virus, and *Mycoplasma pneumoniae* are commonly identified triggering pathogens [16]. Other viruses that may be complicated with GBS include Zika virus and influenza virus [19, 20]. Since the start of the COVID-19 pandemic, many cases of GBS have been reported to be associated with the disease [21].

Variable pathophysiological mechanisms are involved behind different variants of GBS [22]. In the acute motor axonal neuropathy (AMAN) form of GBS, the infecting organisms probably share homologous epitopes with the proteins in peripheral nerves, and therefore, the antibodies produced during infection cross-react with the nerves, causing axonal degeneration [17]. Proposed target molecules in AMAN are likely to be gangliosides expressed on the membrane of motor nerve axons [17]. In the acute inflammatory demyelinating polyneuropathy (AIDP) variant of GBS in which the exact molecular mechanism is not yet understood, the immune system reacts against target epitopes in Schwann cells resulting in demyelination [17, 23].

In acute motor-sensory axonal polyneuropathy (AMSAN), the pathophysiology is similar to that in AMAN, with the target proteins being located on sensory and motor neuronal axons [17, 22]. The pathophysiological mechanism behind the occurrence of GBS in patients post COVID19-related disease could be multifactorial, and depend upon the variant of GBS that would manifest in the affected patient.

GBS classically presents with sensory impairment followed by generalized weakness that develops over several days. In most of the affected patients, it leads to quadriparesis [24]. Approximately two-thirds of patients report upper or lower respiratory tract infection or gastrointestinal illness preceding the development of GBS-associated symptoms [24]. Similarly, up to one-third of patients require intubation and ventilation because of respiratory failure [24]. Our patient rapidly deteriorated during the COVID-19 recovery phase, manifesting with flaccid paralysis including oxygen desaturation due to involvement of respiratory muscles.

In most patients, thorough neurological examination often leads to the suspicion of GBS, as was the case with our patient. Routine biochemical analyses are followed by more revealing diagnostic workup such as cerebrospinal fluid analysis and, most importantly, the neurophysiological assessments that lead towards the diagnosis [15]. Electrodiagnostic studies are not required to diagnose GBS, but they are helpful in establishing the diagnosis, especially in cases that present with atypical features [25]. Typically the electrophysiological studies in GBS reveal sensorimotor polyradiculoneuropathy or polyneuropathy, indicated by reduced conduction velocities, reduced sensory and motor evoked amplitudes, abnormal temporal dispersion, and/or partial motor conduction blocks [25]. A characteristic feature identified by nerve conduction studies is a “sural sparing pattern” in which the sural sensory nerve action potential, primarily in its amplitude, is normal while the amplitude of median and ulnar sensory nerve action potentials is abnormal or even absent [25]. In our patient, while the disease had already entered the recovery phase, symptoms such as proximal muscle weakness and unexplained drop in oxygen saturation led to the suspicion of GBS that was confirmed by electrophysiological studies.

Conventionally, therapeutic options include plasmapheresis, IVIG, in stable patients, while mechanical ventilation is required in patients who develop respiratory failure due to involvement of respiratory muscles [22]. In Afghanistan, healthcare establishments with mechanical ventilator support are rarely available. Similarly, there is no institution to provide plasma exchange services. Therefore, the only option left was IVIG with early commencement of physiotherapy, which had a dramatic effect, resulting in complete resolution of symptoms in a matter of days. This is one of the main reasons our case is being reported, that is, to inform doctors, especially first-line physicians, about this particular complication and thus have lower threshold in initiating the initial screening workup for GBS, including thorough neurological examination.

## Conclusion

In our case report, we presented the rare and most severe variant of GBS, AMSAN, that complicated COVID-19-related disease in its recovery phase. To our knowledge, this was the first case of AMSAN complicating COVID-19. Clinical suspicion and prompt diagnosis led to early intervention, leading to complete recovery. While the world is entering the second wave of COVID-19, clinicians, particularly who manage old-age individuals, need to have insight into this dreadful but manageable complication of COVID-19-related disease.

## Data Availability

All the generated data are included in this article.

## Abbreviations

GBS: Guillain–Barre syndrome
AMAN: Acute motor axonal polyneuropathy
AMSAN: Acute motor-sensory axonal polyneuropathy
AIDP: Acute inflammatory demyelinating polyneuropathy
COVID19: Coronavirus disease 2019
FBG: Fasting blood glucose
HBA1c: Hemoglobin A1c
CREAT: Serum creatinine
BUN: Blood urea nitrogen
ALT: Serum alanine aminotransferase
AST: Serum aspartate aminotransferase
TBILL: Total bilirubin
CBC: Complete blood count
Hb: Hemoglobin
WBC: Total white blood cell count
PLT: Platelet count
PH: Power of hydrogen ion concentration
PCO_2_: Partial pressure of carbon dioxide in blood
PO_2_: Partial pressure of oxygen in blood
HCO_3−_: Bicarbonate ion concentration
SPO^2^: Oxygen saturation
APB: Abductor pollicis brevis
ADM: Abductor digiti minimi
B.Elbow: Below elbow
A.Elbow: Above elbow
AHL: Abductor hallucis longus
EDB: Extensor digitorum brevis
F2: Finger 2
F5: Finger 5
NR: Not recorded
NCV: Nerve conduction velocity
